# Is hypoglycemic polyneuropathy reversible after curative resection of an insulinoma? A case report and literature review

**DOI:** 10.3389/fendo.2026.1924634

**Published:** 2026-08-28

**Authors:** Anna Körei, Barbara Gombos, Katalin Borka, Peter Kempler, Judit Tőke, Miklós Tóth

**Affiliations:** 1 Department of Internal Medicine and Oncology, Faculty of Medicine, Semmelweis University, Budapest, Hungary; 2 Department of Neurology, Nyírő Gyula National Institute of Psychiatry and Addictions, Budapest, Hungary; 3 Department of Pathology, Forensic and Insurance Medicine, Faculty of Medicine, Semmelweis University, Budapest, Hungary

**Keywords:** case report, hypoglycemia, insulinoma, painful neuropathy, peripheral neuropathy

## Abstract

**Introduction:**

Insulinoma is a rare neuroendocrine tumor characterized by episodes of hypoglycemia. Peripheral neuropathy associated with hypoglycemia is an uncommon complication of insulinoma, and its reversibility remains a subject of debate within the literature.

**Case report:**

We describe the case of a 22-year-old female patient diagnosed with a solitary pancreatic insulinoma. The patient experienced recurrent episodes of headache, blurred vision as well as numbness, tingling and burning pain of the limbs over a period of 6 to 12 months prior to definitive diagnosis. Electrophysiological investigations revealed findings consistent with multiple radiculopathies and a motor-predominant axonal sensorimotor polyneuropathy primarily affecting the upper limbs. The biochemical diagnosis of endogenous hyperinsulinemic hypoglycemia was confirmed through a supervised standard 72-hour fasting test. Abdominal computed tomography imaging identified a contrast-enhancing tumor at the pancreatic body-tail transition. The patient underwent a laparoscopic distal pancreatectomy with splenectomy. Postoperative follow-up revealed prompt resolution of hypoglycemia, alongside improvement in dysesthesia and sensory deficit in the extremities as well as most neurophysiological parameters. However, tendon reflexes remained diminished, and subtle distal-predominant lower limb weakness and gait imbalance persisted for several months postoperatively.

**Discussion:**

This case underscores the importance of recognizing a rare neural complication associated with insulinoma. The incomplete resolution of neurological symptoms and neurophysiological abnormalities highlights the potentially lasting impact of recurrent severe hypoglycemia on peripheral neural function.

## Introduction

Insulinoma is a rare neuroendocrine tumor characterized by episodes of hypoglycemia. It represents the most common cause of endogenous hyperinsulinemic hypoglycemia in non-diabetic individuals, with an incidence rate of approximately 1 to 3 cases per million annually. The majority of insulinomas occur sporadically, although familial cases have been associated with multiple endocrine neoplasia type 1 (MEN-1) syndrome. Clinically, insulinomas manifest primarily through hypoglycemic symptoms resulting from excessive insulin secretion, typically precipitated by overnight fasting or physical exertion. Biochemical diagnosis is confirmed via a 72-hour fasting test, followed by tumor localization using imaging modalities such as computed tomography (CT), magnetic resonance imaging (MRI), endoscopic ultrasound, or, more recently, 68Ga-exendin PET/CT. The definitive treatment is surgical removal of the tumor (1, 2).

Peripheral neuropathy resulting from hypoglycemia caused by insulinoma is a rare complication reported in the literature, predominantly associated with prolonged and severe hypoglycemic episodes. Typical clinical features of hypoglycemia-induced peripheral neuropathy secondary to insulinoma include predominantly symmetrical, distal involvement of both sensory and motor nerves. Additionally, there is a tendency for motor and upper limb symptoms and neurophysiological deficits to predominate (3, 4). In many cases, peripheral neuropathy co-occurs with central nervous system manifestations such as convulsions and paroxysmal dystonia (5–7). The majority of patients with insulinoma experience resolution of neurological symptoms following curative surgical intervention. However, literature presents inconsistent data regarding the remission of peripheral neuropathy after complete surgical tumor removal.

This report discusses a case of a young patient presenting with sensorimotor peripheral neuropathy associated with hypoglycemic episodes secondary to insulinoma.

## Case report

The 22 -year-old female patient originally presented to the neurology department with episodes of temporal headaches, blurred vision, diplopia and facial tingling. The general physical examination yielded unremarkable findings. The initial outpatient neurological assessment showed no focal neurological deficits, and a comprehensive ophthalmological evaluation revealed no abnormalities. At that time, no definitive diagnosis was established, and atypical migraine alongside a functional neurological disorder were considered in the differential diagnosis. In laboratory tests, only slightly elevated gamma GT enzyme activity (86 U/L) and below-normal fasting serum glucose values (3.2 mmol/L and 3.5 mmol/L) were of note. Blood count, serum electrolytes, liver and kidney functions were normal.

Four months later, she was re-evaluated due to recurrent visual disturbances accompanied by symptoms including limb numbness, tingling and burning pain as well as calf weakness and headache. The onset of these symptoms followed a severe hypoglycemic episode. On neurological examination, symmetric distal paresthesia was observed affecting all four limbs, with a predominant involvement of the upper extremities, alongside symmetric dysesthesia of both lower extremities extending up to the knees. Vibration and proprioceptive sensations remained intact. Deep tendon reflexes were symmetrically diminished. Mild weakness was evident during weight-bearing, as well as during heel and toe walking. No muscle atrophy was detected.

Based on fasting hypoglycemias, the diagnosis of insulinoma was suspected and the patient was referred to our Department. On admission, the patient’s BMI was 25.8 kg/m^2^). No specific finding on physical examination could be established. A 72-hour fasting test confirmed endogenous hyperinsulinemic hypoglycemia (**Table 1**).

**Table 1:** Blood test results obtained at the time of insulinoma diagnosis.

Parameter	Reference range	Result
Serum glucose at 0 min and 120 min, respectively, during oGTT; mmol/L	<7.8 - <11.1	5.7 - 6.5
HbA1c; %	<6.5	6.0
Results following a 16-hour fasting period during the onset of hypoglycemic symptoms: • serum glucose; mmol/L • serum insulin; µIU/mL • serum C-peptide; ng/mL	3.9-6.1 low low	1.7 36.2 4.95
Sodium; mmol/L	135-145	139
Potassium; mmol/L	3.5-5.1	4.2
Calcium; mmol/L	2.1-2.6	2.55
GFR; ml/min/L	>90	>90
Uric acid; µmol/L	180–480	282
Triglyceride; mmol/L	<1.7	0.9
Cholesterol; mmol/L	<5.2	4.5
GOT; U/L	12–38	29
GPT; U/L	10–40	31
GGT; U/L	5–55	27
ALP; U/L	25–100	59
LDH; U/L	45–200	166
Serum bilirubin (total); µmol/L	2–17	6
Total protein; g/L	60–78	77
TSH; mIU/L	0.4–4.0	1.894
Cortisol; nmol/L (at 08:00 a.m.)	138–635	222
IGF-1; µg/L (female between 21–24 years)	102 – 441	310
Prolactin; µg/L	<25	5.04
Chromogranin A; ng/mL	<19	<16.9
Vitamin B12 level; pmol/L	118–701	543
Folic acid level; nmol/L	6.1-38.5	24.2

For differential diagnosis, common etiologies of acquired polyneuropathy were systematically excluded, including deficiencies in folic acid and vitamin B12, renal and hepatic insufficiency, diabetes mellitus, hypothyroidism, and infectious conditions such as HIV and viral hepatitis. Autoimmune markers — specifically ENA, ANA, and ANCA — tested negative, along with anti-ganglioside and anti-myelin-associated glycoprotein (MAG) antibodies. The clinical progression did not align with typical primary autoimmune polyneuropathies, including Chronic Inflammatory Demyelinating Polyneuropathy (CIDP), Miller-Fisher syndrome, or Guillain-Barré syndrome. Paraneoplastic antibody profiling via immunoblot was negative for titin (MGT30), recoverin, PKC-gamma, GAD65, Zic4, Tr(DNER), SOX1, Ma2, Ma1, Amphiphysin, CV2 (CRMP5), Ri, Yo, and HuD. Serum protein electrophoresis excluded the presence of monoclonal gammopathies. All relevant laboratory results are summarized on **Table 1**.

Based on these findings, the clinical presentation was ultimately attributed to hypoglycemic peripheral neuropathy secondary to insulinoma.

Abdominal computed tomographic scan with iv. contrast revealed a 25-mm well-circumscribed hypovascularized lesion located between the pancreatic body and tail, radiologically fully compatible with a neuroendocrine tumor. Additional investigations did not reveal manifestations of multiple endocrine neoplasia type 1 (MEN-1) syndrome, and genetic testing for MEN-1 syndrome proved negative.

Electroneurography (ENG) studies revealed a combination of chronic inhomogeneous axonal sensorimotor polyneuropathy and multiple radiculopathy. At the lower limb, distal motor latencies and nerve conduction velocities (NCVs) of peroneal and tibial nerves were normal, but the motor amplitudes were moderately decreased. Decreased F-wave persistence and increased latency were found on the left peroneal nerve; all other F-waves were absent. As for the upper limbs, sensory amplitudes of the right radial, ulnar and especially of the median nerve were reduced. Right median nerve showed moderately decreased distal sensory NCV. Ulnar nerve distal motor latency was normal while that of the median nerve was increased. Motor action potentials on the upper limb were reduced, with the NCVs being normal and F-waves missing. Electromyography (EMG) revealed mild fibrillation potentials in the m. common extensor digital and anterior tibial muscle. The latter showed mildly reduced interference pattern and motor unit action potentials consistent with neurogenic lesion (denervation). The results of the ENG investigations are summarized in **Table 2**.

**Table 2:** Nerve conduction study findings of the patient at initial presentation and during postoperative follow-up.

Nerve stimulated	Parameter	Reference value	Preop	Postop 4 months	Interpretation of preop-postop changes
Motor nerves					
Median (right)	CMAP (mV)	≥4.0	2.1*	6.1	Normalized
	Distal latency (ms)	≤4.4	4.9*	4.5*	Improved
	NCV (m/s), elbow–wrist	≥49	47.4*	56.5	Normalized
Ulnar (right)	CMAP (mV)	≥4.0	3.6*	ND	—
	Distal latency (ms)	≤3.3	2.79	ND	—
	NCV (m/s)	≥49	55.0	ND	—
Peroneal (right)	CMAP (mV)	≥4.0	1.22*	3.4*	Improved
	Distal latency (ms)	≤4.8	12.3*	5.1*	Improved
	NCV (m/s), fibular head–ankle	≥44	42.1*	44.3	Normalized
Peroneal (left)	CMAP (mV)	≥4.0	1.14*	1.4*	Stable
	Distal latency (ms)	≤4.8	13.1*	6.0*	Improved
	NCV (m/s), fibular head–ankle	≥44	43*	53.7	Normalized
Tibial (right)	CMAP (mV)	≥4.0	3,9*	7.9	Normalized
	Distal latency (ms)	≤5.8	3,64	6.0*	Worsened
	NCV (m/s), popliteal fossa	≥41	44,7	43.1	Stable
Tibial (left)	CMAP (mV)	≥4.0	1.3*	4.3	Normalized
	Distal latency (ms)	≤5.8	4.08	4.2	Stable
	NCV (m/s), popliteal fossa	≥41	64.9	41.2	Stable
Sensory nerves					
Median (right)	SNAP (µV)	≥10	3.3*	16.7	Normalized
	NCV (m/s)	≥50	43.9*	51.6	Normalized
Ulnar (right)	SNAP (µV)	≥10	5.0*	ND	—
	NCV (m/s)	≥50	53.7	ND	—
Radial (right)	SNAP (µV)	≥15	6.6*	16.8	Normalized
	NCV (m/s)	≥50	48.5*	50,0	Normalized
Sural (right)	SNAP (µV)	≥6	20.9	21.9	Stable
	NCV (m/s)	≥40	45.0	48.3	Stable

CMAP: compound muscle action potential. NCV: nerve conduction velocity. SNAP: sensory nerve action potential. ND: not done. *: abnormal value.s

For the treatment of peripheral neuropathy, vitamin B complex, alpha-lipoic acid therapy, and for alleviating neuropathic pain, pregabalin was initiated. With implementation of two former agents, the patient reported some improvement of motor symptoms. The patient did not undergo any physiotherapy interventions for polyneuropathy.

Diazoxide was initially administered at a dosage of 50 mg twice daily, with an escalation to a total daily dose of 250 mg. By the third day of treatment, the patient remained free of hypoglycemia. Nonetheless, the patient experienced weight gain from 78.0 kg to 84.7 kg. Consequently, after 14 days of diazoxide administration, the treatment was discontinued due to this adverse event.

Afterall, laparoscopic distal pancreatectomy with splenectomy was performed using prophylactic antibiotic coverage and thromboprophylaxis. The postoperative course was without any significant event. Intraoperative findings included a well-encapsulated tumor which was subsequently confirmed as a WHO grade 2 neuroendocrine tumor (NET) with a Ki-67 index of 13%. Immunohistochemical analysis demonstrated positivity for INSM-1 and insulin. Pathological staging indicated successful complete tumor removal (R0) with no perineural (Pn0) or lymphovascular (LV0) involvement, although tumor cells demonstrated vascular invasion (V1). None of the 9 regional lymph nodes were positive for metastatic tumors. The multidisciplinary team suggested regular imaging surveillance for postoperative monitoring.

Four months postoperatively, the patient reported notable improvement in sensory perception and dysesthesia, with complete resolution of paresthesias. Symptomatic medication for neuropathic pain could be withdrawn. Neurological assessment at this follow-up revealed enhanced sensory function and muscle strength; however, deep tendon reflexes remained slightly diminished. Residual deficits included mild distal-predominant lower limb weakness and subtle gait imbalance. characterized by unsteadiness during standing and tiptoe walking. Neurophysiological testing demonstrated overall functional recovery consistent with clinical remission of symptoms. Sensory nerve conduction studies of the median and radial nerves normalized. The median nerve exhibited persistent prolongation of motor latency despite normalized nerve conduction velocity and compound muscle action potential (CMAP). Improvements in peroneal nerve motor action potential amplitude and NCV were evident compared to baseline, though motor action potential values remained below normal limits. The left-sided predominance of abnormalities in the peroneal nerve and right-sided predominance in the tibial nerves persisted, with increased distal motor latencies. (**Table 2**). F-waves were absent on the peroneal nerve, whereas normal F-waves were registered in all other investigated nerves. At one-year follow-up, the patient reported no dysesthesia, no paresthesia, only a slight weakness of the calf during tiptoe walking. At this time, no nerve conduction studies were performed, as they are invasive procedures and the clinical scenario corresponded to almost complete recovery.

## Discussion

We report the case history of a young female with peripheral sensorimotor polyneuropathy as a consequence of hyperinsulinemic hypoglycemia caused by a solitary insulinoma. Peripheral neuropathy constituted one of the initial presenting symptoms and was diagnosed through comprehensive clinical evaluation and neurophysiological assessment.

To date, slightly more than 40 cases of hypoglycemic neuropathy associated with insulinoma have been reported in the literature. Notably, a single case report of insulinoma-induced hypoglycemic polyneuropathy associated with MEN-1 syndrome has been reported (6), otherwise sporadic cases are known. The several recent publications concerning neurological manifestations of insulinoma refer to neuropsychiatric symptoms and seizures (8–11). Additionally, the review by Popa et al. addressing diagnostic challenges of insulinoma focuses solely on autonomic and neuroglycopenic manifestations of hypoglycemia without mentioning peripheral polyneuropathy at all (12).

Insulinoma remains a rare cause of hypoglycemia-induced peripheral neuropathy; most hypoglycemic neuropathy cases evolve in patients with diabetes mellitus treated by insulin or hypoglycemic antidiabetic agents. These cases of hypoglycemic polyneuropathy share features with neuropathy described in association with insulinoma. Conversely, treatment-induced neuropathy in diabetes (TIND) linked to rapid correction of chronic hyperglycemia typically results in neuropathic pain, autonomic dysfunction and allodynia (13).

Common clinical features of hypoglycemic peripheral neuropathy cases associated with insulinoma include symmetrical development and upper limb predominance of symptoms and neurological deficits (3, 4). Generally, sensory and motor nerve fibers are affected with predominance of motor damage (5–7, 14, 15). As few cases were described as pure sensory neuropathy (14), isolated motor neuropathy without any proof of sensory nerve involvement has also been reported (16). Typically, sensory dysfunction presents as positive symptoms of neuropathy such as paresthesia, numbness or burning pain. Although sensory symptoms were uncommonly volunteered in connection with hypoglycemic episodes in an early case series, this symptom could be verified from almost half of the patients upon targeted questioning (4). Interestingly, one case of peripheral polyneuropathy became apparent only after the removal of insulinoma when the more severe neurological deficits improved (4). The latter observation attracts attention to the fact that sensory symptoms of insulinoma-related hypoglycemic neuropathy might be underreported. Clinically meaningful sensory loss is scarcely reported in the literature. Kaul et al. described a case of a 10-year-old girl with attenuation of all sensation modalities in a glove-and-stocking distribution accompanied by areflexia (15).

As for motor neuropathy, manifestations range from wasting and weakness of distal muscles, especially affecting the small muscles of the hands and feet, to foot drop, para- and hemiparesis. Proximal muscle weakness and amyotrophy have been reported but these features are not typical (14, 17–19).

In several cases, the (re)occurrence of neuropathic symptoms are closely linked to the hypoglycemic episodes, i.e. they occur intermittently. In the case series by Dagett et al., both sensory and paralytic symptoms exhibited considerable variability, with paralytic episodes persisting from one hour to three days (4). Other patients experience a slow, gradual worsening of sensorimotor symptoms wherein the onset of symptoms definitely linked to a severe or prolonged episode of hypoglycemia.

Our case presented with distal symmetrical sensorimotor symptoms and neurophysiological alterations that align with typical features of insulinoma-associated hypoglycemic neuropathy described in the literature. Specifically, it was a distal, symmetrical sensorimotor polyneuropathy with sensory symptoms comprising paresthesia and neuropathic pain predominantly affecting the upper limbs. Both clinical and neurophysiological assessments confirmed the predominance of motor and upper limb involvement. In our patient, although motor nerve involvement was obvious on clinical examination and electrophysiologically well-documented, our patient had only subtle clinical motor symptoms compared to some other cases reported (6, 15).

Diplopia and visual disturbances are generally accepted as common symptoms of neuroglycopenia when hypoglycemia deprives the brain its unbearable energy substrate. However, diplopia and blurred vision may also be considered as a consequence of intermittent mononeuropathy of the cranial nerves. Discoordination and weakness of the external ocular muscles may consequently impair eye muscle alignment and focusing in patients with insulinoma.

The scarce neurophysiological investigations available demonstrated decreased motor action potential amplitudes with preserved motor NCVs in patients with hypoglycemic neuropathy secondary to insulinoma. Sensory action potentials and sensory NCVs are usually found to be reduced, which coincides with our findings. Electromyography studies demonstrated consistent denervation and muscle atrophy in previously investigated patients (5), which also agrees with our data. In addition, our patient exhibited absence of F-waves, indicative of radicular involvement. Only a few electrophysiological studies reported hypoglycemic injury of the spinal roots or spinal cord (14, 19).

The reversibility of neuropathy related to insulinoma after surgical removal of the tumor, is still debated. Heckman et al. reported only partial remission of polyneuropathy at nine months after surgery (5), while Vinhosa Bastos et al. observed slow and partial improvement of motor symptoms fifteen months after surgery, despite prompt and complete resolution of hypoglycemia. The latter patient suffered decreased muscle strength, and his ENG/EMG analysis showed signs of electromyographic evidence of chronic neuropathic injury even after 2 years (16). A case of predominant distal motor polyneuropathy showed resolution over a 2-year period (4). Our patient reported a substantial improvement of symptoms as early as four months after successful surgery. Yet, some neurophysiology measures remained pathological. Regarding time span needed for recovery from peripheral polyneuropathy induced by insulinoma, our patient may expect a full recovery of neurological symptoms and alterations in the long term. The rapid resolution of symptoms in our patient compared to literature data may be attributable to the relatively brief interval between symptom onset and surgical cure. Nevertheless, the contribution of neuropathy-targeted medication cannot be entirely excluded. Namely, the majority of reported cases retrospectively associated with insulinoma and neuropathy had symptoms for years. As for the medication, we found only one record on the administration of B12 supplementation for insulinoma-related neuropathy, unfortunately without any improvement (16). Extended follow-up studies are necessary for elucidating the long-term consequences of hypoglycemic polyneuropathy induced by insulinoma.

The pathophysiology of hypoglycemic neuropathy secondary to insulinoma remains poorly understood because of the limited neuropathological evidence available. Muscle specimens from patients who died in hypoglycemic coma have demonstrated denervation and muscle atrophy, while sural nerve biopsies primarily revealed axonal degeneration (3). Experimental studies suggest that only prolonged severe hypoglycemia induces nerve fiber degeneration, predominantly affecting the poorly perfused central fascicular regions of peripheral nerves (20). Large myelinated fibers appear particularly vulnerable. Proposed mechanisms include impaired axonal transport and neuronal energy depletion, together with metabolic dysfunction of Schwann cells and endoneurial microangiopathy (3, 21, 22). In addition, evidence from diabetes indicates that glycemic variability contributes to peripheral neuropathy through mitochondrial dysfunction, endoplasmic reticulum stress, inflammation, endothelial dysfunction, and impaired neurotrophic support (23–26). Although not specifically investigated in insulinoma, recurrent cycles of hypoglycemia and subsequent glucose correction may similarly contribute to the development and progression of peripheral neuropathy.

Our study has several strengths and limitations as well. Noteworthy, we were able to document peripheral sensorimotor polyneuropathy and its almost complete recovery after curative surgery of the insulinoma not only by improvement of clinical symptoms but by comprehensive neurophysiological evaluation as well. Only a few case reports have provided such detailed documentation of the clinical and electrophysiological features of hypoglycemic peripheral neuropathy secondary to insulinoma. The detailed differential diagnostic workup further strengthens our report. Besides, we thoroughly correlated our data with similar cases previously reported. However, a limitation of our report is the fact that the initial and follow-up electrophysiological investigations were performed by two different neurologists at separate institutions, which introduces inter-observer variability.

In conclusion, hypoglycemic peripheral neuropathy is an uncommon but significant complication of insulinoma that characteristically occurs in patients with protracted and severe hypoglycemic episodes. Its typical clinical presentation is distal symmetrical sensorimotor neuropathy with predominant motor nerve involvement. Upper limb predominance of symptoms and neurophysiological deficits is also characteristic. The clinical symptoms of hypoglycemic peripheral neuropathy should prompt the consideration of hyperinsulinemic hypoglycemia due to insulinoma even in the absence of typical symptoms of hypoglycemia (hypoglycemia unawareness). Notably, symptomatic peripheral sensorimotor neuropathy can be the initial manifestation of an insulinoma preceding the development of more severe or more typical symptoms of neuroglycopenia. Although most of the patients with insulinoma experience relief of the neurological symptoms after curative surgery, in many cases, the neurological recovery might be incomplete. Such persistent deficits of hypoglycemic neuropathy may affect patients’ quality of life and underline the critical importance of timely diagnosis and surgical intervention with the aim of preventing irreversible neural injury.

## Data Availability

The raw data supporting the conclusions of this article will be made available by the authors, without undue reservation.
